# Alternative Pathway Inhibition by Exogenous Factor H Fails to Attenuate Inflammation and Vascular Leakage in Experimental Pneumococcal Sepsis in Mice

**DOI:** 10.1371/journal.pone.0149307

**Published:** 2016-02-12

**Authors:** Erika van der Maten, Saskia van Selm, Jeroen D. Langereis, Hester J. Bootsma, Fred J. H. van Opzeeland, Ronald de Groot, Marien I. de Jonge, Michiel van der Flier

**Affiliations:** 1 Laboratory of Pediatric Infectious Diseases, Department of Pediatrics, Radboud Institute for Molecular Life Sciences, Radboudumc, Nijmegen, The Netherlands; 2 Pediatric Infectious Diseases and Immunology, Department of Pediatrics, Radboudumc, Nijmegen, The Netherlands; University of Leicester, UNITED KINGDOM

## Abstract

*Streptococcus pneumoniae* is a common cause of sepsis. Effective complement activation is an important component of host defence against invading pathogens, whilst excessive complement activation has been associated with endothelial dysfunction and organ damage. The alternative pathway amplification loop is important for the enhancement of complement activation. Factor H is a key negative regulator of the alternative pathway amplification loop and contributes to tight control of complement activation. We assessed the effect of inhibition of the alternative pathway on sepsis associated inflammation and disease severity using human factor H treatment in a clinically relevant mice model of pneumococcal sepsis. Mice were infected intravenously with live *Streptococcus pneumoniae*. At the first clinical signs of infection, 17 hours post-infection, mice were treated with ceftriaxone antibiotic. At the same time purified human factor H or in controls PBS was administered. Treatment with human factor H did not attenuate disease scores, serum pro-inflammatory cytokines, or vascular permeability and did not significantly affect C3 and C3a production at 26 h post-infection. Therefore, we conclude that inhibition of the alternative complement pathway by exogenous human factor H fails to attenuate inflammation and vascular leakage at a clinically relevant intervention time point in pneumococcal sepsis in mice.

## Introduction

The Gram-positive pathogen *Streptococcus pneumoniae* is a major human pathogen and one of the most common causes of pneumonia, meningitis and sepsis. Young children, the elderly and immune-compromised individuals are especially at risk to develop invasive pneumococcal disease [1, 2]. Despite the availability of effective antibiotic agents, case fatality rates of pneumococcal sepsis are still high [3]. A major problem in sepsis is the ongoing inflammation and organ dysfunction following the antibiotic treatment [4].

The complement system is a major human defence and clearance system and is highly activated during sepsis [5]. Complement mediated C3 opsonisation and phagocytosis play a vital role in clearance of encapsulated Gram-positive pathogens, such as *S*. *pneumoniae*. Individuals with a deficiency in complement C3 activation or regulation thereof are more susceptible to invasive pneumococcal diseases [6]. Importantly, in the cascade of complement reactions, complement activation products also referred to as anaphylatoxins are released. These anaphylatoxins, C3a and in particular C5a, both promote inflammation via cross talk with Toll-like receptors [7]. Increased generation of complement activation products C3a and C5a has been associated with severity of sepsis [8–10]. Furthermore, increased complement activation has been found to enhance cytokine production, endothelial permeability and cardiac dysfunction [11–14].

The classical pathway of complement activation has long been recognized as the dominant complement activation route in host defence to *S*. *pneumoniae* [15]. The classical pathway is activated by C1q binding to antibody-antigen complexes on the bacterial surface. More recent the significance of the lectin pathway has been recognized [16]. This pathway is activated by binding of ficolin A or collectin 11 to the pneumococcal surface [16]. Importantly, the alternative pathway (AP) amplification loop plays a crucial role in the amplification of the initial activation of the classical and lectin pathway [17]. In the AP, spontaneous low-level hydrolysis of plasma C3 leads to deposition of C3b on the activating surface. Consequently, C3b formed by one of the three pathways can be amplified by the AP amplification loop, initiating a positive feedback loop resulting in enhanced complement activation. The quantitative contribution of AP amplification to classical pathway-induced C5a generation can be up to 80% [18]. This indicates a major contribution of the AP on the release of complement activation products [5].

The AP is closely regulated. Complement factor H (FH) is a key negative regulator of AP activation both in the plasma as well as on the cell surfaces. FH recognizes host cell surfaces and inhibits activity of the C3 convertase to avert injury of host tissues [19]. FH binds to C3b, accelerates the decay of the AP C3 convertase and acts as a co-factor for factor I mediated inactivation of C3b [19–22].

We hypothesize that after the onset of sepsis and initiation of antibiotic therapy, inhibition of AP activation is desirable to ameliorate sepsis associated inflammation and vascular leakage. Moreover, several other animal studies demonstrated the efficacy of adjuvant therapy inhibiting complement activation in animal models of sepsis [23–27]. The aim of our study was to investigate whether inhibition of the AP amplification loop by administering exogenous human FH (hFH) at a clinically relevant time point, at the first onset of clinical symptoms, combined with antibiotic treatment could attenuate inflammation and vascular leakage in a mouse model of pneumococcal sepsis.

## Methods

### Ethics Statement

This study was carried out in accordance with the recommendations of ‘OECD Guidance Document on the Recognition, Assessment, and Use of Clinical Signs as Humane Endpoints for Experimental Animals Used in Safety Evaluation’ (OECD Guidance Document 19, 2000). The protocol was approved by the Animal Ethics Committee of Radboud University, Nijmegen, The Netherlands (Permit Number: 2012–274).

### Animals

Eight weeks old female C57BL/6 (wild-type) mice (n = 36) obtained from Charles River Laboratory were used for the experiment. Mice were maintained in individually ventilated cages under a 12h light/12h dark cycle with controlled temperature (22 ± 2°C) and relative humidity (55 ± 5%). The mice had an average weight of 20.0 gram (± 1.0) before the start of the experiment.

### Bacterial Strains and Growth Conditions

A mouse-passaged *S*. *pneumoniae* strain TIGR4 (serotype 4) was used for infection [28]. The mouse-passaged TIGR4 strain was grown in Todd-Hewitt broth supplemented with 5 g/L yeast extract (THY) or on Columbia blood agar (BA) plates (Becton Dickinson) at 37°C and 5% CO_2_ to an optical density at 620 nm of 0.2 and stored in aliquots at -80°C in 15% glycerol. The number of colony forming units (CFU) per milliliter (CFU/mL) was determined by plating serial 10-fold dilutions on BA plates.

### Experimental Procedure

Mice were randomly divided into 4 groups; infected mice injected intraperitoneally (i.p.) with hFH (n = 10), or with i.p. phosphate buffered saline (PBS) as control treatment (n = 10), uninfected mice were injected i.p. with hFH (n = 6) or i.p. PBS (n = 10) respectively. The study was divided in four experiments to allow sufficient time to perform all the measurements at the end of the experiment. In each experiment 2 or 3 mice from every group were used.

Mice were infected intravenously in the tail vein with 1x10^7^ CFU in 100 μl PBS (control mice received PBS alone). Previous work showed that hFH was able to restore complement C3 levels in homozygous FH deficient mice demonstrating that hFH is functional in mice [29]. Furthermore, it was demonstrated that human FH inhibited cleavage of mouse C3 and mouse factor B in plasma [30]. Expression of human FH completely protected homozygous FH deficient mice from developing kidney abnormalities associated with the loss of FH [30]. We choose to administer 600 μg purified hFH intraperitoneally (i.p) (Complement technologies) (600 μl of the 1 μg/μL purified hFH diluted in PBS) as i.p injection with a volume up to 600 μl was well tolerated. Earlier work documents, that a dose of 500 μg hFH injected i.p. is sufficient to restore complement C3 levels in completely FH deficient mice, demonstrating this dose is effective under extreme conditions [29]. Analogous to previous studies hFH was injected i.p [29]. At the first onset of clinical signs (t = 17 h) mice were injected i.p. with 600 μg hFH in 600 μl PBS (control mice received 600 μl PBS alone). At the same time mice were injected with ceftriaxone 25 mg/kg intramuscular (Fresenius Kabi Nederland B.V.). Antibiotic treatment allowed to examine the effect of hFH modulation of complement activity on the inflammatory response while avoiding differences in bacterial CFU counts due to potential inhibition of bacterial clearance by hFH. At 26 h after infection the experiment was ended and CFU, vascular leakage in the liver, cytokine and complement protein levels were measured as described below. Pilot experiments demonstrated that mice in general did not reach the humane endpoint at this time point.

### Measurement of Disease Score

During the experiment, mice were monitored for clinical signs and disease severity each hour and weighted at t = 0, t = 17, t = 21 and t = 26 hours. Mice were scored according to their condition. The following score was used: *Ruffled coat*: showing signs of a ruffled coat (1); Dull ruffled coat, observed mildly around neck and back (2); Ruffled coat (3); *Hunched back*: Mildly hunched back (2); Hunched back (3); *Reduced mobility*: Less mobile but still being active and reacts to any handling (3); Aberrant and slower movement with back legs (4); Hardly walking, needs to be pushed to get going (5); *Weight loss*: >5% body weight loss from t = 0 (2); >10% body weight loss from t = 0 (3); >15% body weight loss from t = 0 (5); >20% body weight loss from t = 0 (15); *Skin temperature*: <35.5°C (15); *Overall state*: no signs of disease (0); Moribund state *(15)*. Based on the disease score humane endpoints were defined. An overall disease score ≥ 15 was used as a surrogate marker of mortality and animals with a disease score ≥ 15 were killed to minimize animal pain, distress and discomfort.

### Colony Forming Units Count

At t = 26 h, mice were anesthetized with 2.5% (vol/vol) isoflurane over oxygen and blood was collected by sub-mandibular bleeding. Blood was collected in Eppendorf safe lock tubes. Bacteria were recovered from the blood by plating serial dilutions on BA plates. Following overnight incubation of the plates at 37°C, CFU were counted.

### Measurement of Cytokines and Complement Proteins in Mice

Blood samples were kept on ice and were centrifuged at 4°C 10.000 rpm after coagulation of the blood. Serum was collected, aliquoted and stored at -80°C for further analysis. To investigate the immune response in mice infected with *S*. *pneumoniae* TIGR4, the general inflammatory markers interleukin 6 (IL-6) and macrophage inflammatory protein 2 (MIP-2) were measured in post-infectious serum samples using ELISA assays (Mouse IL-6 ELISA, Ebioscience, 88–7064; Mouse CXCL2/MIP-2 Quantikine ELISA Kit, R&D Systems, MM200). In addition, the following complement proteins were measured with ELISA assays; mouse C3a (mC3a) (USCN life science, E90387Mu), mouse C3 (mC3) (Mybiosource, MBS564065) and hFH (Hycult, HK342).

### Measuring Vascular Permeability

Vascular leakage in the liver was measured by the method described by Von Drygalski *et al*., 2012 [31]. Infrared fluorescence measurements (IRF) were performed to determine Evans blue (EB) albumin extravasation to quantify vascular permeability. At t = 26 h, mice were treated under anesthesia with 100 μL of 1% EB dye (Sigma Aldrich) in PBS via retro orbital injection and mice remained under anesthesia. Prior to injection, the EB solution was filter-sterilized (Millex, 0.22 μm; Millipore). At 15 min after the EB injection the abdominal cavity and chest were opened by blunt dissection. The vena cava inferior was visualized and cut through. The liver was flushed via the portal vein with a total volume of 40 mL PBS containing heparin at 120mm Hg, thereby removing the EB dye from the vasculature in the liver, followed by harvesting the liver. Mice remained under anesthesia during the whole procedure until the mice died by the perfusion and cervical dislocation. The liver was weighted and placed in a 6 wells tissue culture plate (Co-star). The liver was scanned using the Odyssey infrared imager (LI-COR, Lincoln, NE, USA) with Application software version 2.1.15, 700 channel, focal plane set at 3 mm and laser intensities set at L1.5. Area intensities from the bottom of each well were recorded as integrated fluorescence intensities (I.I) per well area. After scanning the organ was encircled and raw fluorescence intensities (RFI) were recorded and multiplied by the wet organ weight to estimate the concentration of EB in the organ [31].

### Statistical Analysis

Data of the mice experiments are expressed as median and interquartile range. Difference between mice groups were analyzed using the Mann-Whitney test with a Bonferroni correction in case of multiple comparisons. Differences were considered statistically significant when p < 0.05.

## Results

### Exogenous hFH Fails to Attenuate Disease Scores, Inflammatory Cytokine Production, and Vascular Leakage in the Liver

**Fig 1:**
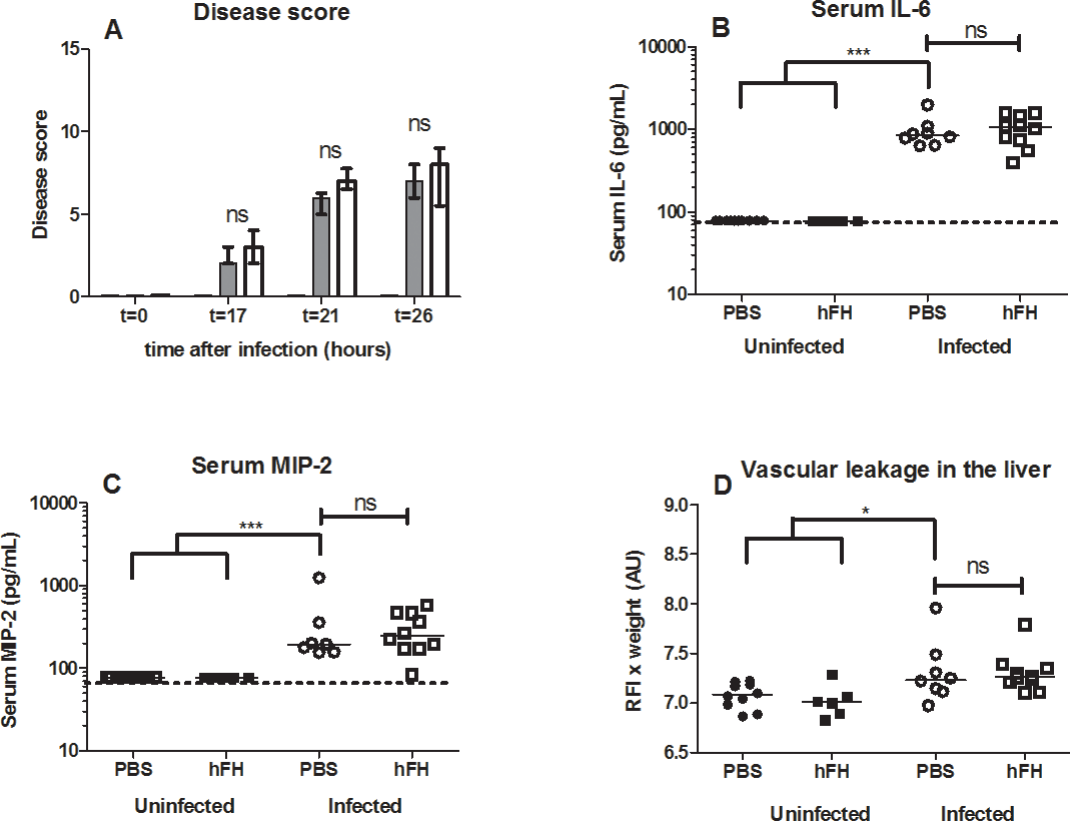
Exogenous hFH fails to attenuate disease scores, inflammatory cytokine production, and vascular leakage in the liver. Mice infected with 1x10^7^ CFU of *S*. *pneumoniae* (TIGR4) and sham infected control mice were all treated with antibiotics at t = 17 h and indicated groups received an injection with hFH or PBS as control (n = 10). The disease score was monitored at t = 17, t = 21 and t = 26 hours after inoculation (A). The black bar represents uninfected mice, gray bar infected control mice and white bar hFH treated mice. Data points represent the median value with interquartile range. At t = 26 h, serum pro-inflammatory cytokines IL-6 and MIP-2 were measured by ELISA (B, C). Liver vascular leakage was measured by Evans Blue-albumin extravasation to quantify vascular permeability (D). Raw fluorescence intensities (RFI) were recorded and multiplied by the wet organ weight to estimate the concentration of Evans Blue in the organ. Each point depicted in graphs B,C and D indicates one mouse. One infected mice of the PBS treated group reached the humane endpoint at t = 22 h and was excluded from the graphs. Furthermore one (IL-6 Fig 1B) respectively two data points (MIP-2 Fig 1C) are missing, as insufficient serum was available. In addition, one data point is missing in the vascular leakage graph, because of a technical failure during injection of EB in one mouse. Cytokine values were analyzed after logarithmic transformation; the horizontal line represents the median. Dash line indicates lower limit of detection. Comparison between groups were performed by using the non-parametric Mann-Whitney test with Bonferroni correction * p < 0.05 was considered significant. ** p< 0.01, *** p<0.001, ns = not significant.

At 17 hours post-infection, before the treatment with antibiotics, the pneumococcal load in the control group and the hFH treatment group were similar (median and interquartile range) control group 5.7x10^5^ (1.6 x10^5^–1.8x10^6^) CFU/mL vs. treatment group 7.1x10^5^ (2.7x10^5^–4.5x10^6^) CFU/mL, *p* = 0.6, Mann-Whitney test. In both groups no viable bacteria were detectable 9 h after antibiotic treatment at termination of the experiment (t = 26 h). The first onset of clinical symptoms of disease occurred at around 17 h post inoculation. The clinical disease score continued to increase rapidly within the first four hours after initiation of antibiotic treatment (t = 21 h) and slowly continued to increase between four and nine hours after initiation of antibiotic treatment (t = 26 h) (Fig 1A). One infected mice of the PBS treated group reached the humane endpoint at t = 22 h. At 26 h after infection clinical disease scores, serum cytokines (IL-6 and MIP-2), and vascular permeability in the liver were all significantly elevated in infected untreated animals compared with uninfected mice (Fig 1B–1D). Treatment with hFH did not attenuate clinical disease scores, serum pro-inflammatory cytokines IL-6 and MIP-2, or vascular permeability in the liver in infected mice (Fig 1B–1D). Exogenous hFH in uninfected control mice had no effect on disease score, serum pro-inflammatory cytokines IL-6 and MIP-2, or vascular permeability in the liver (Fig 1B–1D).

### Exogenous hFH Administered at Onset of Clinical Symptoms Has No Effect on Complement Activation Protein Levels

**Fig 2:**
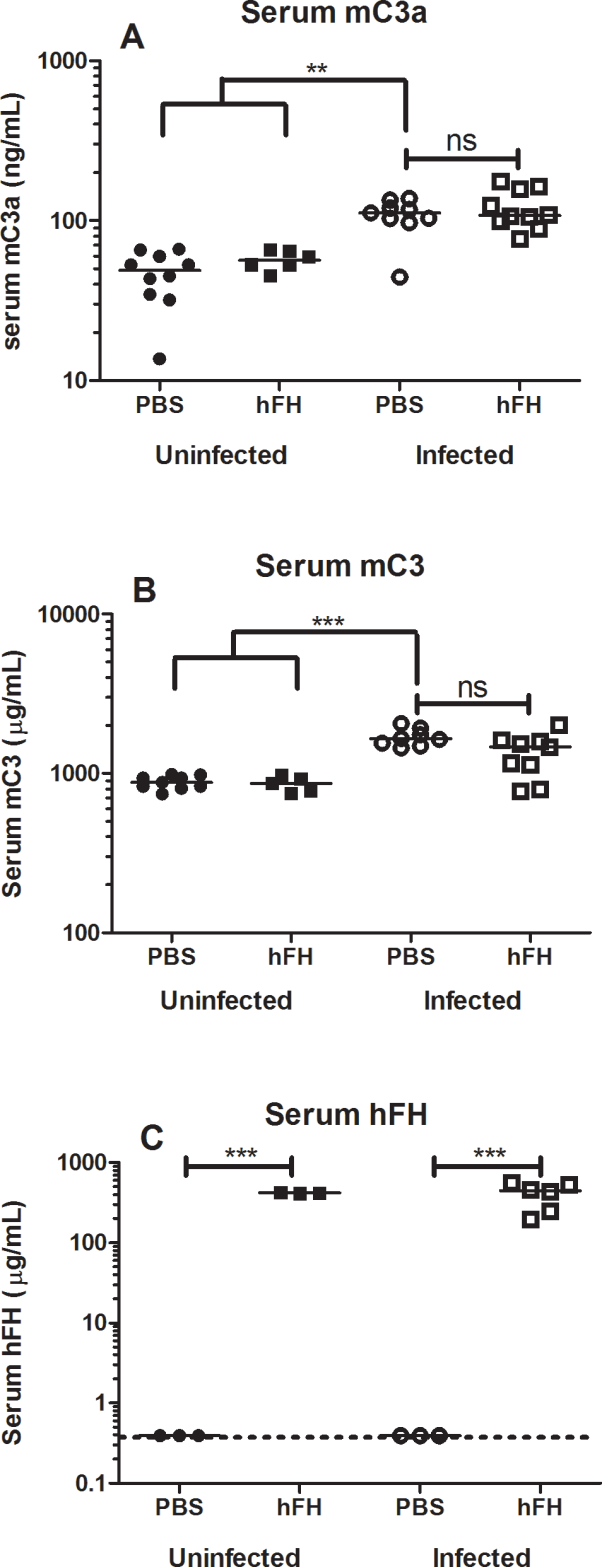
Exogenous hFH administered at onset of clinical symptoms has no effect on complement activation protein levels. Mice infected with 1x10^7^ CFU of *S*. *pneumoniae* (TIGR4) and sham infected control mice, were all treated with antibiotics at t = 17 h and indicated groups received an injection with hFH or PBS as control (n = 10). At t = 26 h serum mC3a, mC3 and hFH levels were measured by ELISA. Each point depicted indicates one mouse. One infected mice of the PBS treated group reached the humane endpoint at t = 22 h and was excluded from the graphs. Two data points of mC3 are missing, as insufficient serum was available to do all the measurements in all mice. Data are individual complement factor values and were analyzed after logarithmic transformation; the horizontal line represents the median. Dash line indicates lower limit of detection. Comparison between groups were performed by using the non-parametric Mann-Whitney test with Bonferroni correction * p < 0.05 was considered significant. ** p< 0.01, *** p<0.001, ns = not significant.

In order to study the effect of exogenous hFH on complement activation, levels of complement proteins were measured. Mouse serum C3a and C3 levels were significantly elevated in the infected mice (Fig 2A and 2B). Exogenous hFH had no effect on mouse C3a and C3 levels. The estimated concentration of hFH present in mice directly after injection is about 400 μg/mL, since a dose of 600 μg intraperitoneal was given to mice with an estimated blood volume of 1,5 mL. The presence of target concentration hFH in treated mice at the end of the experiment was confirmed by ELISA showing high hFH levels (median and interquartile range) 445 (235–534) μg/mL serum at 9 hours after injection (Fig 2C).

## Discussion

It is well established that excessive complement activation contributes to an enhanced inflammatory response and tissue damage in sepsis [12–14, 32]. The amplification mechanism of the AP is believed to play a major role in the release of complement activation products contributing to the overwhelming inflammatory response in sepsis [18]. The main finding in this study is that inhibition of the alternative complement pathway by exogenous hFH at a clinical relevant time point at the first onset of symptoms failed to attenuate inflammation and vascular leakage in a pneumococcal sepsis model in mice.

Complement activation is important in the clearance of endogenous and pathogen derived debris and toxins. Effective clearance of these substances may prevent ongoing induction of inflammation by this debris. In our experimental setting we did not observe increased pro-inflammatory activity resulting from less effective clearance of pathogen debris in mice in which alternative pathway activity was inhibited with FH, however the duration of observation may have been too short to completely exclude this.

In previous studies, it was shown that hFH regulates mouse AP activity [29, 30]. Potentially, the effect of hFH treatment in our study was limited by the timing of administration. hFH was administered at time of clinical symptoms, 17 h after infection, when physiological dysregulation already had occurred. Administration of hFH might have greater potential when given earlier during the course of disease. Attenuation of inflammation and vascular leakage targeting AP activation by exogenous hFH treatment may be more successful in situations where treatment can be administered prophylactic as in cardiopulmonary bypass surgery [33]. However, early hFH treatment at time of infection is clinically not relevant for invasive pneumococcal infection because patients with sepsis will only present with apparent clinical signs and symptoms. Furthermore, others described previously that an abrogated AP activity in mice genetically lacking AP activity enhanced pneumococcal outgrowth and the severity of disease [15, 34]. These studies demonstrate the importance of AP activation in the host defence against pneumococcal invasive infection and early hFH administration may enhance pneumococcal outgrowth.

Enhancement of pneumococcal outgrowth by hFH is especially relevant as the pneumococcal virulence factor pneumococcal surface protein C (pspC) binds hFH, as an immune evasion strategy [35]. This pneumococcal binding capacity is species specific and unique for human FH, and pneumococci do not bind mouse FH [36].

Other studies targeting complement activation by inhibition early in the complement cascade with C1 esterase inhibitor (C1INH) or C3 convertase inhibitor were successful in contrast to our study [25, 26]. C1INH treatment was beneficial on outcome of Gram-negative bacterial sepsis and endotoxin shock in several animal studies [26, 37]. Interestingly, a recent small open label clinical trial described increased survival rates in patients with surgical sepsis treated with C1INH, indicating that intervention might be beneficial in a clinical setting [38]. C1INH has many different anti-inflammatory functions, including non-complement related functions [39]. The beneficial effect of C1INH is therefore not exclusively due to complement inhibition [26].

In addition, early complement inhibition by using compstatin, a C3 convertase inhibitor, was shown protective during *E*. *coli* sepsis in baboons [25]. Interestingly, it was found that complement inhibition was still effective when administered during the second stage of progressive organ failure [25]. Since compstatin blocks the C3 convertase it inhibits all three complement activation pathways and may therefore be more effective in comparison with AP inhibition alone as in the current study.

Our study targeted complement activation, aiming to decrease the activation of complement effector molecules. Potentially it may be more effective to directly block the complement effector molecules such as C3a or C5a. Recently, the potential efficacy of blocking C5a or its receptors in improving outcome in experimental sepsis models was demonstrated. Blocking C5a or its receptors preserved neutrophil function resulting in lower bacterial loads and less severe disease [23, 24, 40]. In cecal ligation and puncture-induced sepsis in rats, it was shown that blocking C5a was even beneficial after the onset of symptoms of sepsis [27].

Complement activity has not only detrimental effects, but is also essential for an effective host defence against invading pathogens such as *S*. *pneumoniae* [14, 41]. Interestingly several novel experimental therapies for sepsis entail stimulating complement activation. Administration of recombinant properdin, a positive regulator of AP activation, resulted in enhanced protection against *S*. *pneumoniae* infection [42]. The use of properdin may be important in the context of antimicrobial resistance in sepsis, however, thus far it has not been assessed whether properdin treatment has an additive effect in combination with antibiotic treatment in sepsis. Additionally, the recombinant properdin used in that study contained a histidine tag [42]. Recently several studies raised concerns about the use of proteins containing a histidine tag, since this may convey anti-microbial activity [43, 44].

To our knowledge this is the first study to assess hFH treatment as adjuvant therapy in sepsis. Our results show that inhibition of the alternative complement pathway by exogenous hFH at a clinical relevant time point at the first onset of symptoms failed to attenuate inflammation and vascular leakage in a pneumococcal sepsis model in mice.
